# Predominance of Intrinsic Mechanism of Resting Heart Rate Control and Preserved Baroreflex Sensitivity in Professional Cyclists after Competitive Training

**DOI:** 10.1371/journal.pone.0148036

**Published:** 2016-01-26

**Authors:** Luciene Ferreira Azevedo, Patricia Perlingeiro, Denise Tessariol Hachul, Igor Lucas Gomes-Santos, Jeane Mike Tsutsui, Carlos Eduardo Negrao, Luciana D. N. J. De Matos

**Affiliations:** 1 Cardiovascular Rehabilitation and Exercise Physiology Unit, Heart Institute (InCor), Medical School of University of Sao Paulo, Sao Paulo, Brazil; 2 Clinical Arrhythmia Unit, Heart Institute (InCor), Medical School of University of Sao Paulo, Sao Paulo, Brazil; 3 Echocardiography Unit, Heart Institute (InCor), Medical School of University of Sao Paulo, Sao Paulo, Brazil; 4 Biodynamic of the Movement of the Human Body, School of Physical Education and Sport, University of Sao Paulo, Sao Paulo, Brazil; 5 Rehabilitation Center, Hospital Israelita Albert Einstein, Sao Paulo, Brazil; Texas A&M University, UNITED STATES

## Abstract

Different season trainings may influence autonomic and non-autonomic cardiac control of heart rate and provokes specific adaptations on heart’s structure in athletes. We investigated the influence of transition training (TT) and competitive training (CT) on resting heart rate, its mechanisms of control, spontaneous baroreflex sensitivity (BRS) and relationships between heart rate mechanisms and cardiac structure in professional cyclists (N = 10). Heart rate (ECG) and arterial blood pressure (Pulse Tonometry) were recorded continuously. Autonomic blockade was performed (atropine—0.04 mg.kg^-1^; esmolol—500 μg.kg^-1^ = 0.5 mg). Vagal effect, intrinsic heart rate, parasympathetic (*n*) and sympathetic (*m*) modulations, autonomic influence, autonomic balance and BRS were calculated. Plasma norepinephrine (high-pressure liquid chromatography) and cardiac structure (echocardiography) were evaluated. Resting heart rate was similar in TT and CT. However, vagal effect, intrinsic heart rate, autonomic influence and parasympathetic modulation (higher *n* value) decreased in CT (*P*≤0.05). Sympathetic modulation was similar in both trainings. The autonomic balance increased in CT but still showed parasympathetic predominance. Cardiac diameter, septum and posterior wall thickness and left ventricular mass also increased in CT (*P*<0.05) as well as diastolic function. We observed an inverse correlation between left ventricular diastolic diameter, septum and posterior wall thickness and left ventricular mass with intrinsic heart rate. Blood pressure and BRS were similar in both trainings. Intrinsic heart rate mechanism is predominant over vagal effect during CT, despite similar resting heart rate. Preserved blood pressure levels and BRS during CT are probably due to similar sympathetic modulation in both trainings.

## Introduction

There are some evidences to suggest that training intensification might substantially influence autonomic control of heart rate (HR) [1–3], arterial blood pressure variability and baroreflex sensitivity (BRS) [1] in highly trained athletes, but the findings are controversial. The intensification happens in macro cyclic competitive training (CT) which combines high intensity and high volume training and is the key component for achieving high performance through the athlete’s training periodization. On the opposite side, macro cyclic transition training (TT) is planned with lower intensity and volume training to offer proper athletes’ recovery [4]. Iellamo et al. [1] had observed higher sympathetic modulation, increased diastolic arterial pressure and blood pressure variability and decreased spontaneous BRS in professional rowers during CT compared with training of lower intensity and volume. However, not all available studies have observed increase on sympathetic modulation with training intensification [2, 5–6]. Differently, elite runners showed higher parasympathetic modulation [2] and higher BRS [5] after intensive training. Additionally, elite bicyclists didn´t show alteration in any parameters of HR variability after intensive training despite a decrease in resting HR [6]. On the one hand CT may change the cardiac autonomic control, on the other hand there is no knowledge related to the influence of different trainings on intrinsic mechanism of HR and its association with cardiac adaptations. Intrinsic mechanism (sinus node change) has also been importantly implicated with HR control in athletes [7–10]. In fact, a classic study showed lower intrinsic HR (IHR) in world-class oarsmen who trained daily and were engaged in national and international rowing competitions compared to control individuals [11]. In addition, we had recently observed that professional cyclists compared to professional runners show higher participation of intrinsic over autonomic mechanism to explain their bradycardia [12] and an association between structural cardiac characteristic and intrinsic alterations, as suggested before [8, 12]. In view of these results, we could suppose that different trainings applied throughout training periodization would influence the intrinsic mechanism of resting HR. Thus, to better understand the influence of 2 distinct trainings inside 1 year training periodization on IHR, autonomic mechanism, blood pressure variability and BRS, we tested the hypothesis that CT exacerbates IHR decrease without increase sympathetic modulation, maintaining blood pressure, blood pressure variability and BRS in professional cyclists. Additionally, we expect an association between echocardiographic parameters and IHR.

## Material and Methods

### Study population

This longitudinal observational prospective study was conducted in healthy professional male road cyclists (N = 10) who were engaged in competitive training for at least 4 years (National Top 5). Of note, this study composes a large study which the first part has been recently published [12]. All athletes were normotensive, non-smokers and had been previously screened for cardiovascular or metabolic diseases. None of the athletes reported use of ergogenic aids or exhibited any overt signs of overtraining [13] according to clinical evaluation and hormone analyses. Each individual signed the informed consent form at the beginning of the assessments. This study complies with the Declaration of Helsinki and was approved by the Scientific and Ethic Committee of the Heart Institute (SDC 2519/04/139) and the Ethics Committee for Analyses of Research Projects (CAPPesq # 929/04) of the Clinical Hospital, Medical school of University of Sao Paulo.

### Study protocol

Athletes were evaluated at the end of TT and at the end of CT (i.e. after the final competition of the year training periodization). The evaluations were carried out during 2 consecutive days, with at least 48 hours apart the last exercise session or the competition for TT and CT, respectively. The athletes did not train during the evaluation days. TT, which is used as a period for performance recovery, lasted 39 ± 8 days and all cyclists stopped cycling for at least 2 weeks. Five cyclists returned to the cycling training sessions after the 2 weeks break. However, they trained an average of 5 days/week; 306 km/week (36% of the total volume of training from the CT) with light to moderate exercise intensity. The others performed walking, jogging or strength exercises with light to moderate intensity. At CT, the athlete’s training routine consisted of 1 daily session, 7 days/week and ≈ 850 km/week (increased volume and exercise intensity – 3 to 4 days/week with high intensity exercise sessions).

### Recorded variables

The continuous ECG sign (modified DII lead) and continuous respiratory rate were obtained (Hewlett Packard M1166A—model 66S—Massachusets, USA). Arterial blood pressure was continuously and non-invasively assessed by a pulse tonometry device (COLIN—7000—San Antonio, Texas, USA). All biological signs were continuously recorded during 15 minutes at rest through the Labele acquisition system by microcomputer (Developed by IT Department at Heart Institute, Sao Paulo, Brazil). The signs were digitalized using an A/D converter 14–16 bit (DI 720, DATAQ Instruments Inc., Ohio, USA) with a sampling of 500 Hz per channel and stored on the hard disk for subsequent analyses.

### HR determination and its mechanisms of control

HR was recorded following 25 minutes of rest in supine position in a calm room with dim light and ambient temperature (22°C to 24°C) after an insertion of a catheter into the athletes’ left arm antecubital vein. Double cardiac blockade was performed to study IHR and vagal effect. Atropine (0.04 mg.kg^-1^) was first administered through the catheter for 2 minutes, followed by 8 minutes of recording, and then esmolol (500 μg.kg^-1^ = 0.5 mg) was infused for 1 minute, followed by 9 minutes of recording. Theses doses have been shown to produce complete blockade of neural cardiac control at rest [14, 15]. Vagal effect was calculated by subtracting resting HR from maximal HR achieved after parasympathetic blockade with atropine. IHR was obtained after completion of infusions (double-blockade).

The autonomic chronotropic influence (AI) at rest [12, 16], the multipliers dependent on the sympathetic (*m*) and parasympathetic (*n*) efferent activities (autonomic modulation) [11, 12, 17] and the autonomic balance (Abal) [10, 12] were calculated and interpreted as previously described.

### Arterial blood pressure, blood pressure variability and BRS

Blood pressure variability analysis has been previously described [18, 19]. Concisely, their harmonic components were evaluated by the autoregressive method (Linear Analysis Program; version 8.5). The following indexes were considered to analyse blood pressure variability: 1) in time domain (standard deviation of systolic and diastolic blood pressure–SDNN) and 2) in frequency domain (total variance and spectral power in the low frequency band: 0.04 to 0.15 Hz, in absolute value). LF components of blood pressure variability are considered to be an expression of vascular sympathetic modulation [18–20].

The analysis of BRS was performed by the sequence method [21, 22] which detects spontaneous oscillations of systolic arterial pressure (SAP) that led to variations of sinus rhythm of HR as previously reported [1, 23]. Both the increase and decrease met the following criteria: 1) RR interval variations > 5 ms; and 2) SAP variation > 1 mm Hg. This method reflects mainly vagally mediated baroreceptor-cardiac responses [24] and has provided reproducible results [25].

### Cardiac structural and functional evaluations

The morphology of the left ventricle (Two-dimensional and Doppler echocardiographic studies by using a cardiac ultrasound machine—HP/Philips Sonos 5500 –Davis Medical Electronics INC., The Netherlands) was assessed and later correlated with IHR. Left ventricle cavity diameters were obtained by two-dimensional echocardiography according to the guidelines of the American Society of Echocardiography and volumes measured according to modified Simpson’s rule [26]. Left ventricular mass, ejection fraction and relative wall thickness were calculated as previously described [12]. All echocardiographic measurements were performed at the same machine and by one experienced observer (J.M.T.). For the determination of intra and inter-observer variabilities, the parameters of the athletes were reassessed by 2 observers (J.M.T and M.M.V.C.). The data are presented as percentage of the mean of two absolute measurements.

### Anthropometry, maximal cardiopulmonary capacity and hormone evaluations

Anthropometric characteristics and maximal cardiopulmonary exercise capacity were assessed as previously described [12]. Blood samples were collected from the athletes’ antecubital vein for the measurement of plasmatic levels of catecholamine, serum cortisol, hemoglobin and hematocrit [12]. Normal levels of norepinephrine, cortisol, hemoglobin and hematocrit were 40 to 268 pg.ml^-1^, 5.4 to 25 μg.dL^-1^, 13.8 to 17.2 g/dl and 42 to 54% (the last two in male adult), respectively.

### Statistical analysis

The sample size was calculated based on the effect of exercise training on IHR in athletes versus non-athletes [11] because we did not find studies evaluating the effect of the season trainings on this variable. The online software (http://www.openepi.com) was used for this purpose with 95% confidence interval (bi-directional), and statistical power of 95%. As a result, the number of 6 patients was considered appropriated. Shapiro-Wilk normality test was applied and all data are parametric, except blood pressure, blood pressure variability and BRS. Parametric data are presented as mean ± SE and non-parametric data as median and interquartile range (IQ). Pared Student *t* test and pared Wilcoxon *t* test were employed to test the differences between TT and CT for parametric and non-parametric variables, respectively. Pearson product-moment correlation coefficients were applied to verify correlations between vagal effect and IHR; and between IHR and echocardiographic parameters. Statistical analyses were performed using Statistica and SPSS softwares. *P* value ≤ 0.05 was set for statistical significance.

## Results

Table 1 shows that anthropometric measurements were similar in both trainings. However, as expected, VO_2_peak was higher in CT. Serum cortisol and hemoglobin levels were similar in TT and CT respectively (cortisol = 11.2 ± 0.8 vs. 11.0 ± 1.1 μg.dL^-1^, *P* = 0.85; hemoglobin = 15.8 ± 0.2 vs. 16.3 ± 0.3 g/dl, *P* = 0.08) and hematocrit levels were higher in CT (45.6 ± 0.6 vs. 48.6 ± 1.1%, *P* = 0.01). However, cortisol, hemoglobin and hematocrit values were within the normal range.

**Table 1:** Age, anthropometric variables and maximal cardiopulmonary capacity in professional cyclists at 2 different trainings.

	Cyclists (N = 10)		
	Transition Training	Competitive Training	*P* value
Continuous time in Competitive Training (years)	9 ± 1		
Age (years)	26 ± 0.9		
Body Fat (%)	9.1 ± 1.9	6.7 ± 1.1	0.06
Lean Mass (kg)	63.7 ± 1.8	64.3 ± 1.7	0.51
BSA (m^2^)	1.87 ± 0.05	1.86 ± 0.04	0.17
VO_2_peak (mL.kg^-1^.min^-1^)	71.8 ± 2.4	78.8 ± 2.3	0.001
VO_2_peak (L.min^-1^)	5.01 ± 0.13	5.35 ± 0.09	0.01

Values are given as mean ± SE. BSA = body surface area; VO_2_ = oxygen consumption.

### HR and autonomic and non-autonomic mechanisms

**Fig 1:**
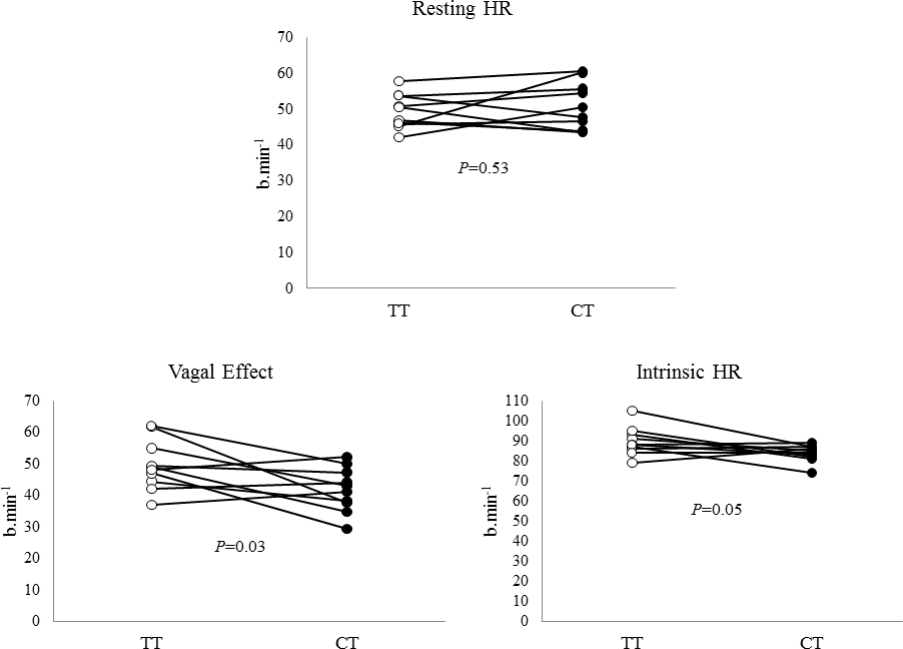
Resting HR, vagal effect and intrinsic HR in professional cyclists (N = 10) at transition (TT) and competitive training (CT). HR = heart rate.

Resting HR was similar in both trainings (TT = 49 ± 2 vs. CT = 51 ± 2 b.min^-1^, *P* = 0.53). All cyclists presented sinus bradycardia (60% between 41 to 50 b.min^-1^ and 40% between 51 to 60 b.min^-1^). Interesting was the fact that cyclists showed lower vagal effect (50 ± 3 vs. 42 ± 2 b.min^-1^, *P* = 0.03) and lower IHR (90 ± 2 vs. 84 ± 1 b.min^-1^; *P* = 0.05) in CT, despite no changes in resting HR (Fig 1).

Table 2 shows additional parameters of autonomic nervous system. Cyclists presented lower AI in CT, possibly due to lower parasympathetic modulation (higher *n* value and lower vagal effect). Sympathetic modulation demonstrated by both *m* multiplier and norepinephrine level was similar in both trainings. The Abal was higher in CT but was still less than 1.0, which represents a parasympathetic predominance. Further analysis showed that there was a correlation between vagal effect and IHR (r = 0.639; *P* = 0.002).

**Table 2:** Autonomic nervous system parameters in professional cyclists at 2 different trainings.

		Cyclists (N = 10)		
		Transition Training	Competitive Training	*P* value
Autonomic Influence (AI) (%)		-45 ± 1.8	-40 ± 2.3	0.03
Multipliers	Parasympathetic modulation (*n*)	0.50 ± 0.02	0.55 ± 0.02	0.05
	Sympathetic modulation (*m*)	1.10 ± 0.02	1.10 ± 0.02	0.93
Autonomic Balance (Abal)		0.55 ± 0.02	0.60 ± 0.02	0.03
Norepinephrine (pg.ml^-1^)		159 ± 40	158 ± 31	0.98

Values are given as mean ± SE.

### Arterial blood pressure, blood pressure variability and BRS

Table 3 shows that both systolic and diastolic arterial pressures were similar during TT and CT. However, SAP variability increased in CT while diastolic arterial pressure (DAP) variability was maintained in the more intensive training. BRS was preserved during CT and respiratory frequency was within the high frequency range.

**Table 3:** Resting arterial blood pressure, blood pressure variability and spontaneous baroreflex sensitivity in professional cyclists at 2 different trainings.

		Cyclists (N = 10)		
		Transition Training	Competitive Training	*P* value
SAP (mm Hg)		122 (116–124)	113 (107–124)	0.44
	SDNN (mm Hg)	3.0 (2.4–3.5)	4.3 (3.0–5.7)	0.06
	Variance (mm Hg^2^)	7.6 (5.5–9.5)	22.7 (17.3–47.0)	0.02
	LF (mm Hg^2^)	(0.4–1.1)	1.4 (1.0–2.6)	0.04
DAP (mm Hg)		62 (59–65)	59 (55–65)	0.31
	SDNN (mm Hg)	3.2 (2.2–3.7)	3.6 (2.9–3.9)	0.19
	Variance (mm Hg^2^)	6.9 (4.5–9.7)	9.1 (6.5–12)	0.16
	LF (mm Hg^2^)	0.8 (0.3–1.8)	0.9 (0.6–1.8)	0.31
BRS (ms.mm Hg^-1^)		33.7 (26.2–35.8)	49.8 (33.7–52.7)	0.24
Respiration (Hz)		0.31 (0.28–0.32)	0.31 (0.28–0.35)	0.31

Values are given as median and interquartile range. SAP = Systolic Arterial Pressure

LF = Low Frequency; DAP = Diastolic Arterial Pressure; BRS = Spontaneous Baroreflex Sensitivity.

### Structural cardiac adaptations and Pearson’s correlations

Structural and functional echocardiographic parameters are shown in Table 4. The significant increase in left ventricular diastolic diameter, septum wall thickness, posterior wall thickness, and left ventricular mass show the already expected combined type of cardiac hypertrophy during CT (eccentric plus concentric). The evaluation of diastolic function showed a higher Doppler filling velocity during late diastole (A wave peak) and higher isovolumic relaxation time during CT. Also, the tissue Doppler showed significant increase in E’ and A’ velocities and E wave deceleration.

**Table 4:** Echocardiographic data in professional cyclists at 2 different trainings.

		Cyclists (N = 10)		*P* value
		Transition Training	Competitive Training	
	LVDD (mm)	53 ± 1	55 ± 1	0.01
	EF (%)	61 ± 1.3	60 ± 1.7	0.77
	SWT (mm)	11 ± 0.2	12 ± 0.2	0.01
	PWT (mm)	11 ± 0.2	12 ± 0.2	0.01
	RWT (mm)	0.41 ± 0.01	0.42 ± 0.01	0.21
	LVM (g)	221 ± 7	259 ± 7	0.002
MF	E wave peak velocity cm.s^-1^	77 ± 4	78 ± 3	0.65
	A wave peak velocity cm.s^-1^	40 ± 3	52 ± 3	0.01
	E/A	2.01 ± 0.1	1.57 ± 0.1	0.09
	Isovolumic Relaxation Time (ms)	76 ± 2	97 ± 5	0.03
SLATD	E´ velocity (cm.s^-1^)	16 ± 1	20 ± 1	0.01
	A´ velocity (cm.s^-1^)	7 ± 0.2	10 ± 1	0.05
	E wave Deceleration (ms)	115 ± 3	153 ± 7	0.004
	S wave velocity (cm.s^-1^)	10 ± 1	10 ± 1	0.79
	CTM (ms)	301 ± 6	311 ± 21	0.41
	PCTm (ms)	96 ± 8	101 ± 6	0.87
	RTm (ms)	70 ± 3	77 ± 6	0.85

Values are given as mean ± SE. LVDD = left ventricular diastolic diameter

EF = ejection fraction; SWT = septum wall thickness; PWT = posterior wall thickness

RWT = relative wall thickness; LVM = left ventricular mass; MF = mitral flow; SLATD = septal and lateral average tissue Doppler.

Intra- and inter-observer variability—Mean differences of intra-observer repeated measurements for aorta, left atrium, left ventricular diastolic diameter and septum were 1.6, 2.4, 1.5 and 1.7%, respectively (*P*>0.28) and showed agreement of 88, 94, 96 and 58% (*P*<0.05), respectively. Mean differences of inter-observer measurements for the same variables were 1.9, 5.3, 1.8 and 0.9%, respectively (*P*>0.48) and showed agreement of 83, 89, 93 and 80% (*P*<0.01), respectively.

Pearson’s correlations showed an inverse relationship between septum wall thickness and IHR (r = -0.589; *P* = 0.01), posterior wall thickness and IHR (r = -0.589; *P* = 0.01) and between left ventricular mass and IHR (r = -0.546; *P* = 0.01).

## Discussion

The present study, in professional cyclists, highlights the role of CT affecting the mechanisms involved in resting bradycardia without changing its level. We demonstrated a conversion from vagal to intrinsic mechanism prevalence after CT in high-performance cyclists. Also, CT did not exacerbate cardiac sympathetic modulation and neither impaired BRS. The lower IHR at the CT seems to be associated with structural cardiac remodeling, such as a combine eccentric and concentric hypertrophy, which is specific of professional cycling sport [27].

The maintenance of the level of resting HR in both TT and CT within one year training periodization may reflect the chronic effect of athlete professional exercise because they were involved in training for several continuously years. Corroborating this data, Portier et al. [2] observed similar resting HR in elite runners during basic as well as CT. Despite the maintenance of resting HR we found important changes related to the mechanisms underlying cyclists’ bradycardia, especially in the non-autonomic mechanism. Previous studies have demonstrated reduction on IHR induced by long-term CT in athletes compared with sedentary individuals [8–11, 28]. Nevertheless, to our knowledge, this was the first study to demonstrate the influence of the season trainings on IHR in professional cyclists. CT produced 18% decrease in age predicted IHR (103 b.min^-1^) [29], while in TT this decrease was of 13%. Interesting to note that our athletes presented similar levels of IHR at CT when compared with world-class oarsmen [11] (84 ± 1 vs. 81 ± 7 b.min^-1^, respectively). The correlation between vagal effect and IHR demonstrates that as lower the IHR lower the vagal effect. This supports the idea of a balance between autonomic and non-autonomic mechanisms regulating resting bradycardia in highly trained cyclists. Interesting is the fact that even with lower parasympathetic modulation during CT (higher *n* and lower vagal effect), these athletes still demonstrate a parasympathetic over sympathetic predominance influencing resting bradycardia, as showed by the Abal value lower than 1.0. Thus, parasympathetic modulation may coexist with some degree of intrinsic change in sinus node in professional cyclists, as reported in runners compared with sedentary individuals [10].

The results of the present study show no increase in sympathetic modulation following CT in cyclists, as demonstrated by similar values of *m* and catecholamine between the two trainings. Furthermore, norepinephrine levels were in the normal range, which rules out the possibility of over sympathetic activation. Differently, Iellamo et al. [1] observed a conversion from vagal to sympathetic predominance with strenuous training in high-performance rowers. We emphasize two important points to explain these controversy findings. First, a possible influence of the rowers’ last exercise session on the variables recorded. This might represent a very important artefact, because a persistent sympathetic activation after an exercise training session can be observed up to 24 hours [3]. Our athletes were evaluated at least 48 hours after their major competition, preventing any short-term effects of the last exercise session. Second, the recording related to the 100% of maximum training load in the study of Iellamo et al. [1] was performed 20 days before the Rowing World Championship. This might have avoided the tapering effect when stress is reduced and performance increased [4, 30]. Our athletes were evaluated at the end of CT because we intended to exclude pre-competition stress, eliminate possible influence on autonomic response as previously observed [31] and evaluate the cyclists’ peak performance.

The precise mechanism underlying the remarkable change in sinus node is out of the scope of the present study. However, a relation with cardiac mechanical effects, such as cardiac dilatation and cardiac hypertrophy observed in athletes has been suggested [8, 11]. Considering the cyclists’ training periodization, we observed a higher physiological cardiac dilatation and hypertrophy after CT when they also presented a pronounced decrease in IHR and significant relationship between septum wall thickness, posterior wall thickness and left ventricular mass with IHR. These results reinforce the theory of mechanical effect to explain IHR changes and may be related to sport modalities that impose both static and dynamic components of exercise [27]. Because of this characteristic, cycling promotes a mixture of eccentric and concentric hypertrophy, which may have implication in decreasing IHR [12]. Going further, D’Souza et al. [32] have showed that training-induced bradycardia is caused by intrinsic electrophysiological changes in the sinus node in rodents with a widespread remodelling of pacemaker ion channels via downregulation of the HCN4 channels that regulate the funny current. This study adds important information regarding molecular mechanisms to understand the sinus node influence in bradycardia.

In our study, we did not observe increase in arterial blood pressure nor BRS impairment. However, only SAP variability increased in CT. Higher blood pressure variability, mainly for low frequency band, is related to higher vasomotor tone and its control by sympathetic activity implying in increased peripheral resistance [18]. However, the maintenance of DAP variability indicates that the cyclists may not have an important commitment of sympathetic vascular control because the low frequency band of DAP variability has been proposed as the most important index of the influence of sympathetic fibers on vascular wall [33]. Facing this result we could suppose that the increase on SAP variability may not be representative of the overall framework of the sympathetic modulation that was maintained during CT as observed by the *m* multiplier and norepinephrine level in this training. Interesting to notice that higher blood pressure levels and worsening in BRS has been observed in rowers presenting higher sympathetic modulation during more intense training [1]. However, this alteration was observed during successive competitions of rowers’ championship.

Our athletes presented higher left ventricular diastolic diameter with concomitant increase in diastolic function in CT. These changes show increased cardiac compliance reflecting the enhanced cardiac adaptation due to high hemodynamic overload imposed by high volume and intensity of training in CT. In the opposite direction, some authors have observed impaired diastolic function in athletes following marathon that persisted until 30 days after the running [34]. However, they evaluated the acute effect of exercise and the study was carried out in amateur athletes. It is important to consider that cardiac dysfunction and injury have been associated with less training [35] that can be common in amateur athletes. We had evaluated the chronic exercise effect (training) and based on this, we could suppose that professional athletes whom follow a systematic training periodization might be protected and even prepared for supporting successive high volume and intensity of exercise.

## Conclusions

The present study provides evidence that season trainings influence the mechanisms of resting bradycardia in professional cyclists. CT compared with TT leads to non-autonomic predominance on the resting HR control and keeps preserved BRS and cardiac function. In addition, the specific structural cardiac adaptations may be related to the intrinsic mechanism of resting bradycardia.

## Study Limitation

The main limitation in our study is the fact that sympathetic effect was not studied by autonomic cardiac blockade because our cyclists showed resting HR as low as 42 b.min^-1^ and we choose to preserve their safety. However, we can infer minor sympathetic influence because the athletes showed similar plasma norepinephrine levels in both trainings. The possibility of a doping status cannot be completely ruled out. However, despite not performing anti-doping test in the athletes, we could at least in part exclude doping related to the increase in red blood cells which seems to be common in endurance athletes. We support this assumption based on the normal values of hemoglobin and hematocrit observed at both evaluations. Finally, we only included men in our study, restricting our findings to male cyclists.
